# PERCEIVED SOCIAL ISOLATION IS CORRELATED WITH BRAIN STRUCTURE AND COGNITIVE TRAJECTORY IN ALZHEIMER’S DISEASE

**DOI:** 10.1093/geroni/igac059.2797

**Published:** 2022-12-20

**Authors:** Ye Zhang, Yasuko Tatewaki, Yingxu Liu, Naoki Tomita, Yumi Takano, Taizen Nakase, Tatsushi Mutoh, Yasuyuki Taki

**Affiliations:** Tohoku University, Sendai, Miyagi, Japan; Institute of Development, Aging and Cancer, Tohoku University, Sendai, Miyagi, Japan; Institute of Development, Aging and Cancer, Tohoku University, Sendai, Miyagi, Japan; Institute of Development, Aging and Cancer, Tohoku University, Sendai, Miyagi, Japan; Institute of Development, Aging and Cancer, Tohoku University, Sendai, Miyagi, Japan; Institute of Development, Aging and Cancer, Tohoku University, Sendai, Miyagi, Japan; Institute of Development, Aging and Cancer, Tohoku University, Sendai, Miyagi, Japan; Institute of Development, Aging and Cancer, Tohoku University, Sendai, Miyagi, Japan

## Abstract

Perceived social isolation was associated with future cognitive decline and increase risk of Alzheimer’s disease (AD). However, the impacts of perceived social isolation depending on different clinical stages of AD have not been elucidated. This study was to investigate the influence of perceived social isolation, or loneliness on brain structure and future cognitive trajectories in patients who are living with or are at risk for AD. 176 patients (mean age of 78 years, 39 subjective cognitive decline [SCD], 53 mild cognitive impairment [MCI], 84 AD) underwent structural MRI and neuropsychological testing. Loneliness was measured by one binary item question “Do you often feel lonely?”. Voxel-based morphometry was conducted to evaluate regional gray matter volume (rGMV) difference associated with loneliness in each group. Subgroup analysis was performed in 51 patients with AD (n=23) and pre-dementia status (SCD-MCI, n=28) using the longitudinal scores of Alzheimer’s Disease Assessment Scale-cognitive component-Japanese version (ADAS-Jcog). Whole brain VBM analysis comparing lonely to non-lonely patients revealed loneliness was associated with decreased rGMV in bilateral thalamus in SCD patients, and in the left middle occipital gyrus and the cerebellar vermal lobules Ⅰ-Ⅴ in MCI patients. Annual change of ADAS-Jcog in patients who reported loneliness was significantly greater comparing to these non-lonely in SCD-MCI group, but not in AD group. Our results indicate that perceived social isolation, or loneliness, might be a comorbid symptom of patients with SCD or MCI, which make them more vulnerable to the neuropathology of future AD progression.

